# Complete Genome Sequences of Six Human Bocavirus Strains from Patients with Acute Gastroenteritis in the North Region of Brazil

**DOI:** 10.1128/genomeA.00235-18

**Published:** 2018-04-26

**Authors:** Aripuanã Sakurada Aranha Watanabe, Adriana Luchs, Élcio Leal, Flavio Augusto de Pádua Milagres, Shirley Vasconcelos Komninakis, Danielle Elise Gill, Márcia Cristina Alves Brito Sayão Lobato, Rafael Brustulin, Rogério Togisaki das Chagas, Maria de Fátima Neves dos Santos Abrão, Cassia Vitória de Deus Alves Soares, Xutao Deng, Ester Cerdeira Sabino, Eric Delwart, Antonio Charlys da Costa

**Affiliations:** a Department of Parasitology, Microbiology and Immunology, Federal University of Juiz de Fora, Juiz de Fora, Minas Gerais, Brazil; b Enteric Disease Laboratory, Virology Center, Adolfo Lutz Institute, São Paulo, São Paulo, Brazil; c Institute of Biological Sciences, Federal University of Pará, Belém, Pará, Brazil; d LIM/46, Faculty of Medicine, University of São Paulo, São Paulo, São Paulo, Brazil; e Secretary of Health of Tocantins, Palmas, Tocantins, Brazil; f Federal University of Tocantins, Palmas, Tocantins, Brazil; g Public Health Laboratory of Tocantins State (LACEN/TO), Palmas, Tocantins, Brazil; h Faculty of Medicine of ABC, Postgraduate Program in Health Science, ABC Foundation School of Medicine, Santo André, São Paulo, Brazil; i Retrovirology Laboratory, Federal University of São Paulo, São Paulo, São Paulo, Brazil; j Institute of Tropical Medicine, University of São Paulo, São Paulo, São Paulo, Brazil; k Blood Systems Research Institute, San Francisco, California, USA; l Department of Laboratory Medicine, University of California San Francisco, San Francisco, California, USA

## Abstract

Human bocavirus (HBoV) is commonly associated with acute respiratory tract illness and gastroenteritis. We report six complete genomic sequences of HBoV strains from patients with gastroenteritis in Belém do Pará and Tocantins in the North Region of Brazil. Phylogenetic analysis indicated that the six HBoV strains belong to genotypes 1, 2, and 3.

## GENOME ANNOUNCEMENT

Human bocavirus (HBoV) (family Parvoviridae, subfamily Parvovirinae, genus Bocaparvovirus) is a nonenveloped single-stranded DNA virus composed of four distinct species, HBoV1 to -4 (1–3). The genome size is approximately 5.3 kb, with three open reading frames (ORFs) encoding two nonstructural proteins, NS1 and NP1, and the two structural proteins VP1 and VP2 (4). HBoV1 was first described in children with respiratory infections, while HBoV-2 to HBoV-4 are most commonly found in fecal specimens (1–4). However, there is controversy concerning the actual role of HBoV as both a causative agent of respiratory illness and as a gastrointestinal pathogen, mainly because HBoV is often detected together with other agents (5, 6). Here we report six complete HBoV genome sequences isolated from patients suffering from gastroenteritis in the North Region of Brazil.

The BRA/TO-40, BRA/TO-243, BRA/TO-57, BRA/TO-142, and BRA/TO-237 samples were collected in 2014 in Tocantins State, and the BRA/PA-160 sample was collected in 2015 in Belém do Pará, in Pará State. We performed a deep-sequencing experiment that was an adaptation of different protocols described by Charlys da Costa et al. (7). Twenty to 50 g of human fecal sample was diluted in 500 μl of Hanks' buffered salt solution (HBSS), added to 2-ml tubes with lysing matrix C (MP Biomedicals, USA), and homogenized in a FastPrep-24 5G homogenizer (MP Biomedicals). The homogenized samples were centrifuged at 12,000 × *g* for 10 min, and 300 μl of the supernatant was then filtered through a 0.45-μm-pore-size filter (Merck Millipore, Billerica, MA, USA). The filtrates were treated with a mixture of nuclease enzymes to digest unprotected nucleic acids. Viral nucleic acids were extracted using the Zymo Research ZR-96 viral DNA/RNA kit (Irvine, CA, USA), and cDNA synthesis was performed with avian myeloblastosis virus (AMV) reverse transcription (Promega, Inc., Madison, WI, USA). Second-strand cDNA synthesis was performed using a DNA polymerase I large (Klenow) fragment (Promega, Inc., Madison, WI, USA). Subsequently, a Nextera XT sample preparation kit (Illumina, Inc., San Diego, CA, USA) was used to construct a DNA library. The library was deep-sequenced using the HiSeq 2500 sequencer (Illumina, Inc.).

Three distinct genotypes of HBoV could be identified. The maximum likelihood tree (inferred using FastTree [http://www.microbesonline.org/fasttree]) indicated that three HBoV samples belong to genotype 3 (BRA/TO-40, BRA/TO-243, and BRA/TO-57), two HBoV samples (BRA/TO-142 and BRA/PA-160) belong to genotype 1, and one HBoV sample (BRA/TO-237) belongs to genotype 2. There is relatively limited sequence information about Brazilian HBoV strains at the complete genome level. The data obtained from this study will contribute to the growing database on the molecular diversity of HBoV strains circulating worldwide, as well as contribute to the development of new sensitive and specific molecular diagnostic methods.

### Accession number(s).

The virus genome sequences were deposited in GenBank under accession numbers MG953829 (HBoV-1/BRA/PA-160/Brazil/2015), MG953830 (HBoV-1/BRA/TO-142/Brazil/2014), MG953831 (HBoV-2/BRA/TO-237/Brazil/2014), MG953832 (HBoV-3/BRA/TO-57/Brazil/2014), MG953833 (HBoV-3/BRA/TO-40/Brazil/2014), and MG953834 (HBoV-3/BRA/TO-243/Brazil/2014).
